# Elevated total and direct bilirubin are associated with acute complicated appendicitis: a single-center based study in Saudi Arabia

**DOI:** 10.1186/s12893-023-02258-2

**Published:** 2023-11-10

**Authors:** Mohammed S. Alfehaid, Ayman M. Babiker, Abdullah Hamad Alkharraz, Hamad yousef Alsaeed, Ali Abdullah Alzunaydi, Adi Abdulaziz Aldubaiyan, Hanan Abdalla Sinyan, Bshayr K. Alkhalaf, Rakan Alshuwaykan, Rehana Khalil, Osama Al-Wutayd

**Affiliations:** 1 Department of Surgery, Unaizah College of Medicine and Medical Sciences, Qassim University, Unaizah, Saudi Arabia; 2 King Saud Hospital, Ministry of Health, Unaizah, Saudi Arabia; 3 Research unit, Unaizah College of Medicine and Medical Sciences, Qassim University, Unaizah, Saudi Arabia; 4 Department of Family and Community Medicine, Unaizah College of Medicine and Medical Sciences, Qassim University, Unaizah, Saudi Arabia

**Keywords:** Observational study, Appendicitis, Bilirubin, Direct bilirubin, WBCs, Saudi Arabia

## Abstract

**Background:**

Appendicitis is the most common abdominal surgical emergency and up to our knowledge no previous studies have been conducted in Saudi Arabia particularly at Qassim region and this study aimed to determine a total and direct bilirubin as a predictor of acute complicated appendicitis.

**Methods:**

Observational retrospective study that included patients admitted under the general surgery department with a diagnosis of acute appendicitis at King Saud Hospital, Unaizah, Saudi Arabia. Data on age, gender, BMI, diabetes mellitus, total and direct bilirubin, AST, ALT, sodium, and WBCs levels were obtained.

**Result:**

Among the overall study population of 158 patients, the age median [IQR] was 24.5 [19–31], males were 99 (62.7%), and complicated appendicitis was 33 (20.9%). The multivariable analysis revealed that both elevated total and direct bilirubin are associated with complicated appendicitis (aOR = 3.79, 95% CI: 1.67–8.48, P = 0.001) and (aOR = 4.74, 95% CI: 2.07–10.86, P < 0.001) respectively. A receiver operating characteristic curve showed the best cutoff value of total and direct bilirubin as ≥ 15 µmol/L and ≥ 5 µmol/L respectively, with a sensitivity of 57.6%, and specificity of 73.6% for elevated total bilirubin, and a sensitivity of 54.6%, and specificity of 80% for elevated direct bilirubin.

**Conclusion:**

Elevated total and direct bilirubin are associated with acute complicated appendicitis in this setting. However, it should be supportive factor for acute complicated appendicitis and not considered as standalone diagnostic test.

## Introduction

Acute Appendicitis is the most common surgical emergency which is caused by an obstruction of the lumen, and secretion of fluids into the obstructed lumen which may lead to ischemia, complicated by gangrene or abscess, and may further be complicated by peritonitis and perforation [1]. The chances of perforation increase proportionally with delayed presentation and thus the earlier the appendectomy the fewer chances of perforation occurring [2]. A greater frequency is observed during the second through fourth decades of life, but the diagnosis of appendicitis can be made at any age [3]. Diagnosis of acute appendicitis account for less than 5% among children younger than five years old, while about 40% among the age-group between 10 and 29 years [4]. The lifetime risk in the US is 8.6% in males and 6.7% in females and complicated appendicitis was found intra-operatively to be from 16.5 to 24.4% [5]. Detecting acute appendicitis in some patients with atypical clinical presentation is challenging and further investigations are required for early diagnosis [6]. Perforated appendicitis is associated with poor prognosis which increases morbidity and mortality [7]. The perforated appendicitis in adults is observed in 17 to20% of cases [8]. On other hand, patient may have normal appendix and undergo appendectomy which may have potential complications, hospitalization, and costs [9]. A closed loop formed from appendix lumen obstruction, either by mucosal edema or fecolith. Yet, a paper by Sisson et al. demonstrated that before the obstruction happens superficial mucosal ulcers appear earlier in the disease [10, 11]. Usually, the flora of the appendix contains anaerobes in 60% of the cases, with E. coli and Bacteroides fragilis being the most commonly isolated bacteria in appendicitis [12, 13]. Almost 80% of liver blood supply is from the portal venous system, which transfers bacteria and toxins along with absorbed substances from the intestine. Detoxification of toxins and some of the bacteria may fail due to the high load of bacteria in cases of complicated appendicitis, which may lead to hepatocyte damage hence, a rise in serum bilirubin level. Additionally, macrophages release cytokines including tumor necrosis factor-α (TNF-α) and interleukin-1,6 (IL-1,6) which lead to the inhibition of canalicular excretion of conjugated bilirubin [13–15]. A variety laboratory tests along with other factors may be used to predict appendicitis and complications such as high bilirubin, CRP, Positive peritoneal signs, fever, and old age [16–21]. Total and direct hyperbilirubinemia have been considered as potential biomarkers for acute complicated appendicitis. Bakshi and Mandal found a raised total bilirubin levels among 91.42% of complicated appendicitis cases [22]. In another study by Veeresh Kumar Ireddy et al., 84.21% had elevated bilirubin levels [23]. Sreeramulu et al. also reported that hyperbilirubinemia, particularly with high direct bilirubin levels, may be considered a key marker for predicting gangrene/perforation in appendicitis [24]. To our knowledge, there is no previous study conducted in Saudi Arabia particularly Qassim region, and therefore this is the first study that aimed to assess an association between total and direct bilirubin level and acute complicated appendicitis.

## Materials and methods

### Study setting

The study was conducted at the general surgery department, King Saud Hospital (KSH) in Qassim region. KSH is the second largest hospital in Qassim region with a capacity of approximately 300 beds [25]. The hospital is situated in Unaizah city, the second largest city in Qassim region, one of Saudi Arabia’s 13 administrative regions with over 1.4 million people [26].

### Study design and patient recruitment

Observational retrospective study was conducted at KSH in Qassim region. Medical files of all patients operated for appendicitis in between March 2019 to March 2021were reviewed. All the files of patients aged 14 years and above who had a confirmed appendicitis were reviewed. Patients who were less than 14 years, had missing data, history of cholelithiasis, hepatotoxic drugs, hemolytic or hepatic conditions were excluded. Data on age, gender, BMI, direct and total bilirubin, liver function tests (LFTs), Sodium, and WBC, and intraoperative findings were extracted. After extracting the data using a checklist by one author, another author reviewed the data to ensure the validation.

### Definitions and measurements

The patients were classified according to complicated or uncomplicated appendicitis based on radiological image or gross appearance intra-operatively. Complicated appendicitis includes perforated appendix and/or periappndicular abscess formation while uncomplicated include the Presence of inflammation in the appendix without perforation or abscess formation. The unit of measurement of the total and direct bilirubin was uMol/L and reference range for normal total and direct bilirubin was 5.1–17 uMol/L and 1.7–5.1 uMol/L respectively. However, elevated total and direct bilirubin were ≥ 15 µmol/L and ≥ 5 µmol/L, respectively according to `the optimal cutoff points. The unit of Aspartate transaminase (AST) and Alanine transaminase (ALT) was IU/L, and white blood cells (WBCs) was 10^9^/L.

### Statistical analysis

Statistical analysis was carried out using STATA version 17. Shapiro-Wilk test was used to test if continues variables follows a normal distribution. The mean and standard deviation (SD) were presented for continuous data that were normally distributed and the median and interquartile ranges [IQRs] for that were not normally distributed. Variables with p value < 0.20 in bivariate analysis were included in multivariable analysis. A multiple logistic regression analysis was performed to determine predictors associated with complicated appendicitis. An area under the receiver operating characteristic curve (AUC) was performed and the optimal cut-off points, sensitivity, specificity, positive predictive value, and negative predictive value were reported. Furthermore, simple linear analysis was performed to determine total and direct bilirubin as dependent continues variables and complicated appendicitis as independent variable. Adjusted odds ratio (OR) with 95% confidence interval (CIs) were reported. A p value ≤ 0.05 was considered strong evidence against the null hypothesis.

## Results

A total of 158 patients were included in the study with a median [IQR] age of 24.5 [19–31] years, ranged from 14 to 71 years, and 99 (62.7%) were male. Regarding gross appearance intra-operatively, 33 (20.9%) had complicated appendicitis. The median of total and direct bilirubin was higher among complicated appendicitis and was statistically significant, also elevated total and direct bilirubin was statistically significant, while the following variables; age, BMI, AST, ALT, WBCs, sodium, gender, diabetes mellitus were not statistically significant, detailed in (Table 1). In multivariable analysis, elevated total and direct bilirubin association remained statistically significant for complicated appendicitis (aOR = 3.79, 95% CI: 1.67–8.48, P = 0.001) and (aOR = 4.74, 95% CI: 2.07–10.86, P < 0.001), respectively, detailed in (Table 2). A simple linear regression showed a positive association between total and direct bilirubin with complicated appendicitis (beta coefficient = 11.86, 95% CI: 7.33–16.38, P < 0.001) and (beta coefficient = 3.59, 95% CI: 2.01–5.18, P < 0.001) respectively, detailed in (Table 3). The sensitivity, specificity, PPV, NPP, and AUC of elevated total bilirubin were 57.6%, 73.6%, 36.5%, 86.8%, and 69% respectively and the sensitivity, specificity, PPV, NPP, and AUC of elevated direct bilirubin were 54.6%, 80%, 41.9%, 86.9%, and 72%, respectively, detailed in (Table 4).

**Table 1:** Characteristics of the study population and comparison between complicated and uncomplicated appendicitis (n = 158)

Variable	Complicated appendicitis (n = 33)	Uncomplicated appendicitis (n = 125)	P value
	**Median [IQR]**	**Median [IQR]**	
Age	30 [19–36]	24 [19–30]	0.365
BMI	25.7 [22.8–29.8]	25.6 [23.6–28.3]	0.822
Total bilirubin	15.3 [10–29.1]	9.7 [7.1–15.2]	< 0.001
Direct bilirubin	5.3 [3.4–7.5]	3.3 [2.2–4.5]	< 0.001
AST	19.9 [14.2–25.2]	18.2 [15.7–20.5]	0.316
ALT	22.1 [14.9–31]	19.7 [13.3–25]	0.118
WBCs*	15 (4.4)	13.7 (4.5)	0.145
Sodium*	140 (3.8)	139 (2.6)	0.692
	**Frequency (%)**	**Frequency (%)**	
Gender			
Male	21 (21.2)	78 (78.8)	0.896
Female	12 (20.3)	47 (79.7)	
Diabetes mellitus			
Yes	3 (23.1)	10 (76.9)	0.839
No	30 (20.7)	115 (79.3)	
Elevated total bilirubin			
Yes	19 (36.5)	33 (63.5)	0.001
No	14 (13.2)	92 (86.8)	
Elevated direct bilirubin			
Yes	18 (41.9)	25 (58.1)	< 0.001
No	15 (13)	100 (87)	

*Mean (SD), IQR [Q1–Q3].

**Table 2:** Multivariable analyses of predictors associated with acute complicated appendicitis among the participants

	**Multivariable analysis**		
	**aOR**	**95%CI**	**P value**
Elevated total bilirubin	3.76	1.67–8.48	0.001
WBCs	1.04	0.95–1.15	0.354
ALT	1.02	0.99–1.06	0.214
	**Multivariable analysis**		
	**aOR**	**95%CI**	**P value**
Elevated direct bilirubin	4.74	2.07–10.86	< 0.001
WBCs	1.04	0.95–1.14	0.378
ALT	1.02	0.99–1.06	0.224

**Table 3:** Simple linear regression for total and direct bilirubin associated with complicated appendicitis

Total bilirubin	Beta coefficient		95% CI		P value
Complicated appendicitis	11.86		7.33–16.38		< 0.001
Direct bilirubin					
Complicated appendicitis	3.59		2.01–5.18		< 0.001

**Table 4:** Diagnostic efficacy for elevated total and direct bilirubin

	Sensitivity		Specificity		PPV		NPP	
	**% (95% CI)**		**% (95% CI)**		**% (95% CI)**		**% (95% CI)**	
Elevated total bilirubin	57.6 (49.9–65.3)		73.6 (66.7–80.5)		36.5 (29-44.1)		86.8 (81.5–92.1)	
Elevated direct bilirubin	54.6 (46.8–62.3)		80 (73.8–86.2)		41.9 (34.2–49.6)		86.9 (81.7–92.2)	

## Discussion

In this study of hospitalized subjects, 20.9% had complicated appendicitis. Upon comparing two studies conducted on same objectives in the years 2017 and 2018, it can be inferred that our finding is in agreement with a retrospective cohort study by Serres SK et al. (2017), Which demonstrated a complication rate of 23%, but much lesser than the rate of 67.7% reported by Valon A. Zejnullahu et al., among the patients with acute appendicitis [27, 28]. The authors identified delayed referral by primary care centers and limited access to the medical services as possible reasons behind the higher rate of complicated cases in aforementioned study [28]. Complicated appendicitis may lead to worse outcomes, and its preoperative identification has a profound impact on timing of surgery [29]. Recent advances in ultrasonography and CT have reduced the rate of negative outcomes by assisting in diagnosis of acute appendicitis. The diagnostic significance of sonography with a sensitivity of 85% and specificity of 92% for acute appendicitis has been widely acknowledged, particularly for pregnant ladies and pediatric patients [30, 31]. Furthermore, the helical CT is proven to be more efficient in terms of accuracy than sonography, with sensitivities ranging from 90 to 99% and specificities ranging from 91 to 99% [32–34]. However, Pritchett et al. has recently demonstrated that the utilization of CT scan for diagnosis of acute appendicitis not only results in the increased healthcare cost and a prolonged stay in the emergency department, but also leads to delay in the surgical procedure [35]. Taken that into account, physical examinations and laboratory tests are still of fundamental importance in the diagnostic process [36]. Numerous studies have analyzed the role of elevated bilirubin as a laboratory marker in the diagnosis of acute appendicitis [37–44]. Recently, some researches have also attempted to investigate a possible link between elevated bilirubin and acute complicated appendicitis [27, 45–50]. Based on our investigation, the findings are association of elevated total and direct bilirubin with acute complicated appendicitis. Similar findings were reported by studies conducted by Valon, A et al., Motie MR et al., and Chaudhary et al., that high levels of serum total bilirubin and direct bilirubin were observed in the patients afflicted with complicated appendicitis [27, 51, 52]. Our observations also coincide with several other past studies which also demonstrated a significant association in this regard [27, 38, 45, 53–55]. Elevated bilirubin in cases of appendicitis occurs due to bacteraemia or endotoxaemia, which may be seen in both uncomplicated and complicated appendicitis, though the latter is more commonly associated with this phenomenon [56–58]. We also evaluated sensitivity, specificity, PPV, and NPP of elevated total and direct bilirubin, and found a low sensitivity (total = 57.6%, and direct = 54.6%) and high specificity (total = 73.6%, and direct = 80%) with positive and negative predictive values of 36.5%, and 41.9% for total and 86.8%, and 86.9% for direct elevated bilirubin respectively. Our findings are in agreement with a meta-analysis which also demonstrated a low sensitivity of 49% and a specificity of 82% [41]. Regarding the diagnostic efficiency of elevated bilirubin, our results differ considerably from other studies. Valon A and colleagues showed the sensitivity of total bilirubin as 54.4%, specificity at 72.3%, positive predictive value at 80.4%, and negative predictive value at 43.1% for complicated appendicitis [27]. A prospective study by Chaudhary et al., exhibited a sensitivity and specificity of 100% and 92.9%, respectively, in gangrenous appendicitis, with high positive and negative predictive values of 72.7% and 100%, respectively [52]. Shuaib A et al., observed in a retrospective single-center study a sensitivity of 65.5% and specificity of 75.4%, while PPV and NPV were 28.4%, and 93.6% respectively [59]. The report by Khan and colleagues revealed an 88% sensitivity and 80% specificity in predicting the occurrence of perforative appendicitis [60]. Moreover, D’Souza et al. have proposed in a study that patients with acute appendicitis and complicated appendicitis cases have exhibited high specificity in hyperbilirubinemia [39]. Studies by Beltran MA et al.,. and Panagiotopoulou I et al., concluded showing no diagnostic value of hyperbilirubinemia in the prediction of perforated appendicitis [61, 62]. Looking at the role of the personal preoperative parameters revealed that median age of participants with complicated appendicitis in our study was 30 [18–39], which is in line with the average age of 30.7 years reported in the study conducted in Kosovo [28]. Our study participants were predominantly male like previous studies [45, 46]. However, we didn’t find any association of age and gender with acute complicated appendicitis unlike the previous studies which indicated a significant relationship between old age and male gender, and acute complicated appendicitis [28, 45, 63]. In the current study, multivariate analysis demonstrated no significant association of elevated WBC with acute complicated appendicitis, in contrast to the study conducted in Turkey [45]. However, earlier studies have also revealed that significance of an elevated count of white blood cells (WBC) as predictor of perforated appendicitis is low, and it cannot be considered as specific for appendicitis [37, 38, 47]. Leukocytosis and shift to the left, which were once believed to be an essential laboratory diagnosing criteria for appendicitis with high sensitivity as per Alvarado scoring, are no longer applicable [54, 64]. Our data showed no association of alanine aminotransferase (ALT) and aspartate aminotransferase (AST) with acute complicated appendicitis. Previous reports about the significance of ALT and AST in the diagnosis of acute appendicitis and prediction of perforated appendicitis are very limited and inconclusive [52, 65]. We found no association between BMI and acute complicated appendicitis. No previous studies in our literature review revealed any such association except one study by Ozkan A et al., which reported that in patients with a high BMI (more than 30), the rate of diagnosis of acute appendicitis was delayed and identification of perforated appendicitis was significantly higher as compared to their normal or low BMI counterparts. The authors believed that the possible reason behind delayed diagnosis could be excessive subcutaneous adipose tissue which may diminish the physical examination and imaging methods’ sensitivity [63]. Our study found no association between sodium level and acute complicated appendicitis. However, there is a significant evidence of strong association between hyponatremia and increased risk of complicated appendicitis in literature [29, 59, 66, 67]. The possible reason behind our finding maybe due to the fact that majority our study participants were adults and hyponatremia is found to be more strongly associated with complicated appendicitis among pediatric patients but this partially diminishes in older patient cohorts [68]. Our data do not support the use of elevated total and direct bilirubin as a discrete diagnostic marker of complicated appendicitis. Nonetheless, it would be of more significance as a marker when combined with other diagnostic markers [41, 62]. These diagnostic biomarkers usually have high specificity but low sensitivity, therefore when they are applied in combination, specificity increases but sensitivity remains low [51]. Therefore, patient’s clinical presentation and symptomatic history should be used to support these bio markers rather than determining differentials. Additional studies are required to carry out a thorough analysis of the role of elevated total and direct bilirubin as a plausible diagnostic indicator for acute complicated appendicitis in every age range. While clinical diagnosis at emergency department (ED) in our setting categorized 10 cases as complicated appendicitis, yet only 2 cases had complicated appendicitis. However, elevated total and direct bilirubin were already found in 19 (57%) and 18 (54%) cases, respectively out of 33 as final diagnoses. This may indicate that total and direct bilirubin may help in predicting complicated appendicitis at ED settings in suburban area. The current findings of clinical diagnosis are limited by small number of clinically suspected cases as complicated appendicitis and larger study is required. There are some limitations of current study. The sample size was small which limits the power of the study, and there are inherent limitations of this study due to the retrospective method in a single hospital.

## Conclusions

Elevated total and direct bilirubin are associated with acute complicated appendicitis in this setting. However, it should be supportive factor for acute complicated appendicitis and not considered as standalone diagnostic test.

## Data Availability

The datasets used during the current study are available from the corresponding author on reasonable request.
